# Comparison between automated and manual segmentation in computed tomography for body composition analysis

**DOI:** 10.1186/s12938-026-01535-4

**Published:** 2026-02-20

**Authors:** Cintia Pereira Kuss, Leandra Ulbricht, Klaus Schumacher, Wagner L. Ripka

**Affiliations:** 1 Federal University of Technology–Paraná, Graduate Program in Biomedical Engineering, Av. Silva Jardim, 589, Curitiba, Brazil; 2 Albert Einstein Hospital-Imaging Department, São Paulo, Brazil

**Keywords:** Automated body composition analysis, Comparison of medical image segmentation methods, Computed tomography

## Abstract

**Background and objectives:**

Body composition (BC) assessment by computed tomography (CT) at the level of the third lumbar vertebra (L3) is considered the gold standard for evaluating muscle and adipose compartments. However, manual segmentation is time-consuming and impractical for large data sets. This study aimed to compare the automated method, Identificação Automatizada da Composição Corporal (IACC), with manual segmentation performed using 3D Slicer.

**Methods:**

This retrospective cross-sectional study included 126 participants, each contributing one single axial CT slice at the L3 level, obtained from routine clinical CT scans acquired between November 2023 and January 2025. Manual segmentations were performed in 3D Slicer by a trained evaluator and validated by an experienced radiologist. The IACC automatically identified the L3 level and segmented skeletal muscle (MUSCLE), subcutaneous adipose tissue (SAT), and visceral adipose tissue (VAT). Analyses included intra- and inter-rater reproducibility (ICC, Dice coefficient), Pearson correlation, agreement (ICC), and Bland–Altman plots.

**Results**

The mean analysis time was approximately 5 min per scan with IACC, compared with 25–30 min using the manual method. Intra-rater reproducibility was excellent (ICC > 0.99), and inter-rater agreement demonstrated high Dice coefficients for SAT (0.982), skeletal muscle (0.940), and VAT (0.932). Concordance between methods was high for SAT (ICC = 0.932) and VAT (ICC = 0.958), and good for skeletal muscle (ICC = 0.784), all with Pearson’s r > 0.97. Bland–Altman analysis showed minimal mean bias for VAT (− 0.35 cm^2^) and small variability for SAT, indicating excellent agreement for adipose tissue compartments. In contrast, skeletal muscle presented greater disagreement, with a larger negative bias and wider limits of agreement, reflecting higher variability in automated measurements.

**Conclusions:**

IACC demonstrated high accuracy and efficiency for automated segmentation of SAT and muscle, substantially reducing analysis time compared to manual segmentation. Although greater variability was observed for skeletal muscle measurements, the tool shows promise for use in large-scale population studies and potential clinical applications, particularly, where rapid and standardized analyses are required.

## Introduction

Body composition (BC) assessment is important for monitoring various clinical conditions, such as sarcopenia (loss of muscle mass), obesity, and cardiovascular diseases [1, 2]. Analysis of muscle mass and adipose tissue has already been correlated with survival in breast cancer patients, making it a potential prognostic biomarker [3]. In studies on COVID-19, abnormalities in BC, such as sarcopenia and cachexia (loss of adipose tissue and muscle mass), were associated with worse clinical outcomes [4]. Although indirect methods for BC assessment, such as bioelectrical impedance analysis, are widely used, computed tomography (CT) has emerged as a more accurate and promising alternative [2]. CT enables precise imaging of regions of interest for BC evaluation, from which quantitative metrics can be extracted to measure tissue areas and volumes [5]. It is well-established in the literature that the third lumbar vertebra (L3) level is the most representative region for estimating the distribution of different body tissues, and that cross-sectional areas of skeletal muscle and adipose tissue can be reliably assessed from a single CT slice [6–8].

In Brazil, CT has already been applied for BC analysis, particularly in academic research, including oncology and sarcopenia studies [4]. However, its use remains limited to these contexts, as manual or semi-automated segmentation is time-consuming and impractical for routine clinical practice [8]. To address these limitations, several automated and open-source software packages have been developed, such as Comp2Comp [9]. These tools incorporate deep learning models that allow for automated segmentation and measurement of tissues of interest in BC analysis, making the process more accessible and feasible for both clinicians and researchers. However, they are not sufficient for fully automated workflows. For instance, Comp2Comp does not perform automatic detection of the L3 level, requiring prior identification or integration with another solution before segmentation [10–13].

The lack of nationally validated automated tools restricts the use of CT for BC evaluation. To address this gap, this study aims to validate the Identificação Automatizada da Composição Corporal (IACC), a software developed to segment and quantify body tissues in large volumes of CT examinations. The system is designed to enable faster, reproducible analyses applicable to both population-based research and clinical support [14]. Validating automated software is essential to ensure reliability, accuracy, and clinical applicability [11, 15, 16], as well as to facilitate the use of such tools in population studies for establishing consistent normative values across different conditions and populations. Therefore, the primary objective of this study is to assess the agreement of IACC in CT image segmentation by comparing its results with manual segmentations performed in 3D Slicer.

## Results

A total of 129 participants were initially screened. After exclusions due to artifacts or incomplete visualization of the L3 level, 126 participants (72 women and 54 men) were included in the final analysis, with a mean age of 55.5 years (range: 24–86 years). For each participant, only one axial CT slice at the L3 vertebral level was analyzed. The mean analysis time using the IACC software was approximately 5 min per scan, whereas manual segmentation performed by Evaluator 1 in 3D Slicer required 25–30 min per scan, demonstrating greater efficiency of the automated method.

Intra-rater reproducibility was assessed in 14 randomly selected participants (approximately 10% of the sample). The repeated measurements were performed after an interval of approximately 1 month. Intra-rater reproducibility of the manual method, performed by Evaluator 1, was excellent for SAT, VAT, and muscle, with ICC values close to 1.0, indicating high consistency between repeated measurements. This analysis was conducted using duplicate segmentations of 14 randomly selected images to assess the stability and precision of manual segmentation before using them as the reference standard for comparison with IACC. The results confirmed that Evaluator 1 maintained high fidelity in repeated measurements, ensuring that manual segmentations could reliably serve as the gold standard for validating the automated method.

Table 1 presents the detailed intra-rater ICC values for each tissue type evaluated.

**Table 1:** Reproducibility and agreement analysis for tissue area measurements (cm^2^) between manual and automated (IACC) methods and intra-rater assessment

Tissue compartment	Manual method reproducibility
	**ICC Intra-rater** (IC 95%)
Subcutaneous Adipose Tissue (SAT)	0.999 (0.998–1.000)
Muscle (MUSCLE)	0.999 (0.997–0.999)
Visceral Adipose Tissue (VAT)	1.000 (0.999–1.000)

Inter-rater agreement between human evaluators was assessed using the Dice coefficient, comparing Evaluator 1 (trained) with the reference radiologist (gold standard) in duplicate segmentations (14 participants), as shown in Table 2. This analysis was performed exclusively to ensure that Evaluator 1 was qualified to conduct the manual segmentation of the 126 participants, thus serving as a reliable reference for comparison with IACC. Excellent agreement was observed for SAT (0.982 ± 0.020), muscle (0.940 ± 0.032), and VAT (0.932 ± 0.028), confirming high reproducibility between the specialist and the trained evaluator and the consistency of manual segmentations used as the reference standard.

**Table 2:** Dice coefficient between trained evaluator and gold standard radiologist (gold standard) across body compartments

Tissue compartment	DICE (mean ± DP)
Subcutaneous Adipose Tissue (SAT)	0.982 ± 0.020
Muscle (MUSCLE)	0.940 ± 0.032
Visceral Adipose Tissue (VAT)	0.932 ± 0.028

The comparison between IACC and manual segmentation using 3D Slicer revealed excellent agreement for SAT and VAT, with ICC values of 0.932 and 0.958, respectively, and good agreement for muscle (ICC = 0.784) (Table 3). Pearson’s correlation analysis showed a strong linear relationship between automated and manual measurements: SAT (r = 0.986; p < 0.001), muscle (r = 0.982; p < 0.001), and VAT (r = 0.978; p < 0.001).

**Table 3:** Reproducibility and agreement analysis for tissue area measurements (cm^2^) between manual and automated (IACC) methods–126 participants

Tissue compartment	Manual method reproducibility–ICC (IC 95%)	Agreement: IACC vs. manual–ICC (IC 95%)
SAT	0.929 (0.90–0.95)	0.932 (0.90–0.95)
MUSCLE	0.739 (0.54–0.84)	0.784 (0.71–0.84)
VAT	0.958 (0.94–0.97)	0.958 (0.94–0.97)

**Fig. 1:**
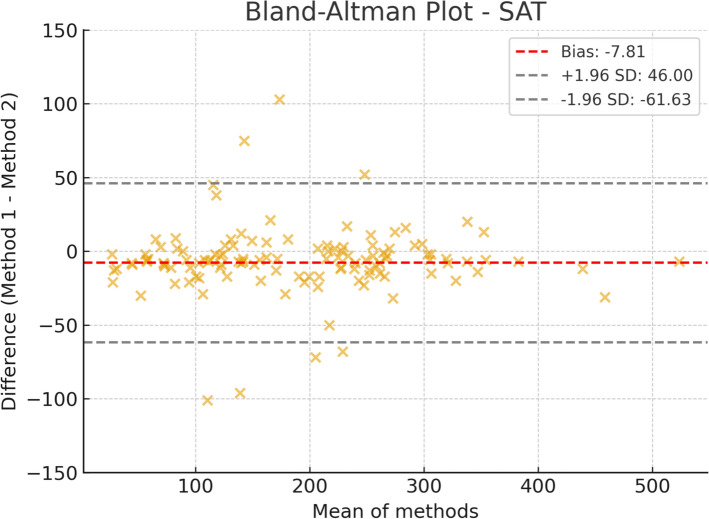
Bland–Altman plot for subcutaneous adipose tissue (SAT) area, comparing automated segmentation (IACC) with manual segmentation (3D Slicer). The plot shows good agreement between the methods. Most data points lie within the ± 1.96 SD limits of agreement. This suggests that the automated method provides consistent results relative to manual segmentation, with small differences that appear randomly distributed, without any relevant systematic trend

**Fig. 2:**
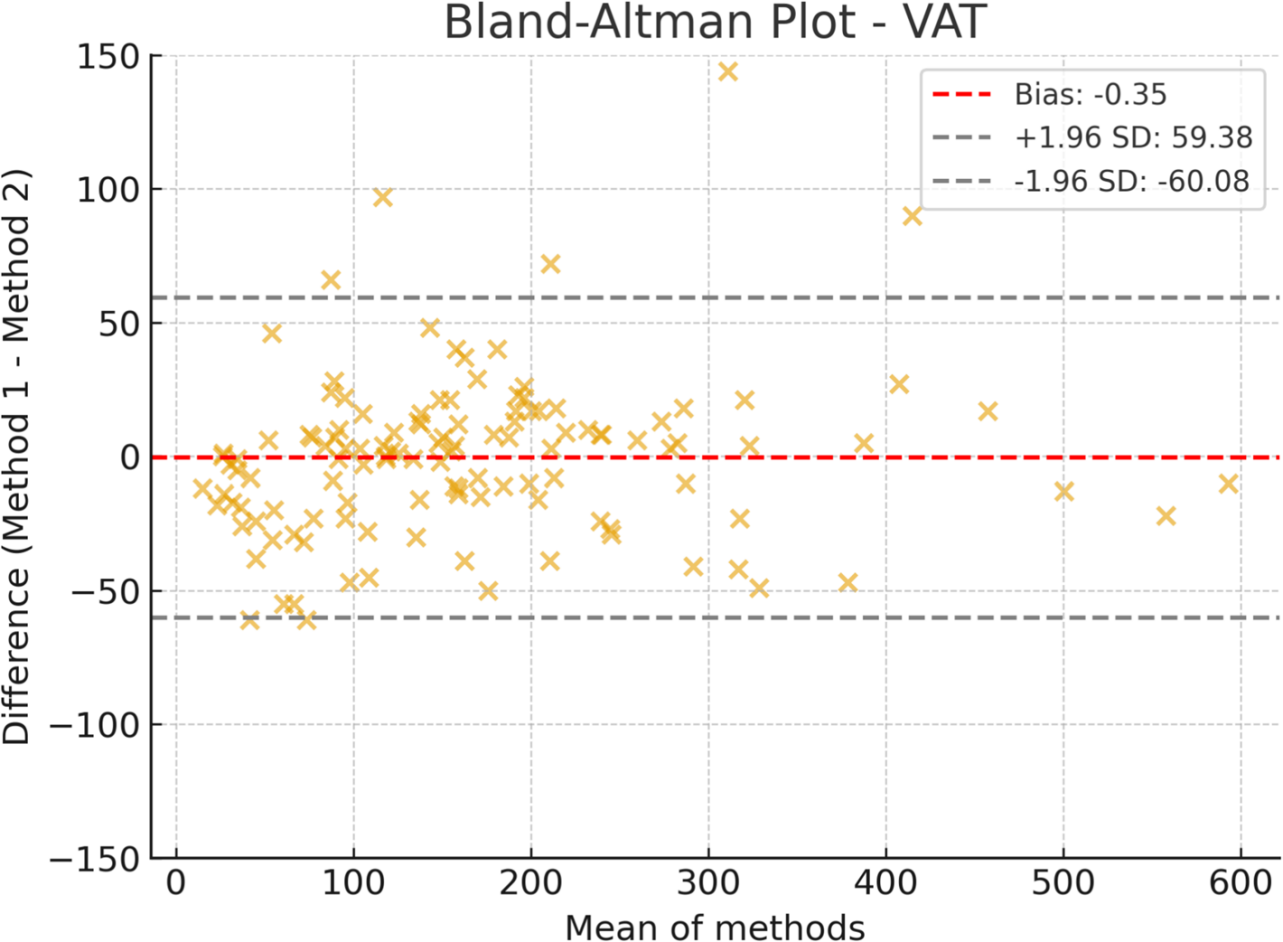
Bland–Altman plot for visceral adipose tissue (VAT) area, comparing automated segmentation (IACC) with manual segmentation (3D Slicer). VAT showed greater dispersion of differences compared with SAT. Nevertheless, most observations remained within the limits of agreement, indicating that the automated method is acceptable, although with slightly higher variability. This may reflect the greater difficulty of segmenting this region, which has less well-defined boundaries

**Fig. 3:**
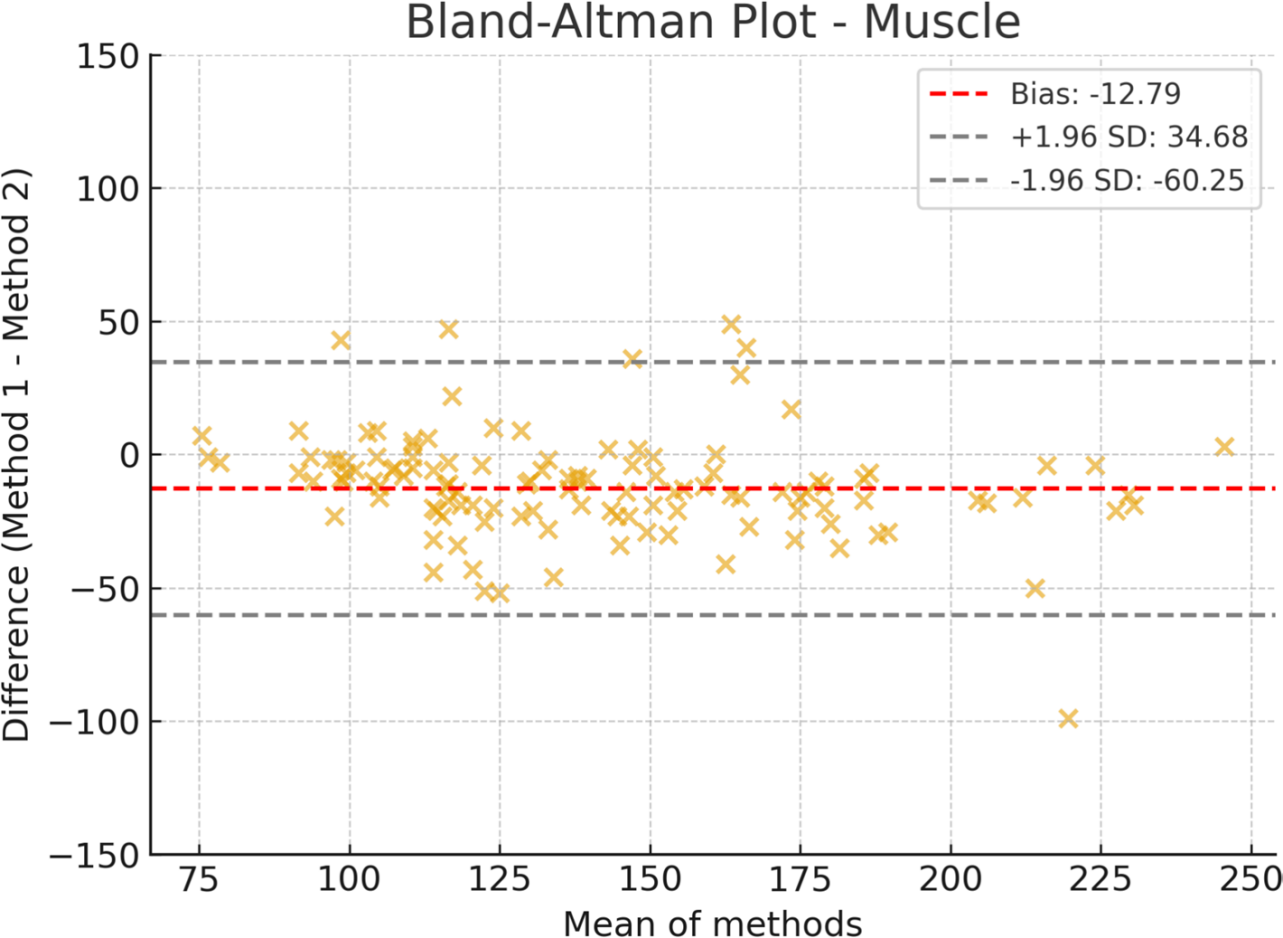
Bland–Altman plot for skeletal muscle area, comparing automated segmentation (IACC) with manual segmentation (3D Slicer)

Figures 1, 2 and 3 present the Bland–Altman plots comparing the automated and manual segmentation methods for body composition measurements. These plots illustrate the agreement between the two approaches, showing the mean difference (bias) and the limits of agreement across the range of measured values. The visual assessment supports the evaluation of systematic differences and the consistency of the automated method in relation to the manual reference.

The agreement analysis between IACC and 3D Slicer revealed tissue-specific differences. For subcutaneous adipose tissue (SAT), the mean bias was – 7.81 cm^2^, indicating a slight tendency for underestimation by IACC. The 95% limits of agreement ranged from – 61.63 cm^2^ to 46.00 cm^2^, with most data points distributed near the bias line, demonstrating good overall agreement and the absence of clinically relevant systematic bias.

For visceral adipose tissue (VAT), the mean bias was – 0.35 cm^2^, essentially negligible, highlighting strong equivalence between the methods. The 95% limits of agreement were –60.08 cm^2^ to 59.38 cm^2^. Despite the reduced bias, greater dispersion of points within this range suggests increased variability for this parameter, reinforcing the inherent complexity of visceral segmentation.

For skeletal muscle, the mean bias was – 12.79 cm^2^, indicating slight underestimation by IACC. The 95% limits of agreement ranged from – 60.25 cm^2^ to 34.68 cm^2^, and although the distribution of points was relatively concentrated, greater dispersion was observed compared with SAT. Nevertheless, the agreement between methods can still be considered satisfactory.

Taken together, these findings demonstrate that the automated method (IACC) shows the best agreement for SAT, whereas skeletal muscle represents the most challenging parameter for automated segmentation, with greater bias and dispersion, while VAT maintains minimal bias but with higher variability.

## Discussion

This study aimed to evaluate the agreement between two CT image segmentation methods at the L3 level for body composition (BC) analysis: IACC, an emerging automated analysis tool, and manual analysis using 3D Slicer, a widely adopted semi-automated segmentation software in research settings. Analyses were conducted with retrospectively collected data from routine clinical practice, a scenario that, even with eligibility criteria applied, preserved the inherent variability of real-world population characteristics and image quality [19]. Rather than being a limitation, this heterogeneity increases the external validity of the study and supports extrapolation of the results to diverse clinical and institutional contexts [20, 21].

### Segmentation performance and technological comparison

The segmentation performance of IACC demonstrated strong equivalence with neural network architectures already established in the literature, reinforcing its position within the current state of the art [22, 23]. The Dice coefficient, a critical spatial overlap metric, revealed that IACC’s performance was comparable to that of other architectures across three of the four compartments analyzed. In line with the literature, Arayne et al. [11] showed that the U-Net architecture can achieve processing times of less than 1 s per image. Similarly, Jang et al. [24] demonstrated that CDFNet is capable of automatically identifying the L3 vertebra and segmenting body compartments with relative differences of < 3.5% compared with manual methods. Since IACC also performs automated L3 identification and presented differences of similar magnitude, these findings reinforce its technological equivalence and viability as an advanced tool.

### Operational efficiency and clinical relevance

Beyond technological equivalence, the processing time analysis of IACC emerged as a key strength. With an average processing time of approximately 5 min per image, the system represents a substantial operational gain compared with manual segmentation, which typically requires 25–30 min per scan according to the literature [10, 18]. This efficiency is particularly promising for large-scale population studies, where standardization and rapid analysis are imperative. Automated tools reduce inter-rater variability and accelerate analysis in large institutional databases, national registries, and multicenter studies, facilitating the secondary use of clinical data for research.

### Bland–Altman agreement and implications

Despite the high correlations and comparable segmentation performance, Bland–Altman agreement analysis revealed that consistency between methods was not uniform across all compartments. The value findings are consistent with those reported in studies comparing automated and manual methods, suggesting no clinically significant systematic bias. The high clustering of data points around the bias line further supports the reliability of IACC for these parameters.

For visceral adipose tissue (VAT), a minimal mean bias (− 0.35 cm^2^) was observed, indicating excellent agreement between IACC and 3D Slicer. The greater dispersion of data, reflected by the 95% limits of agreement (– 60.08 to 59.38 cm^2^), suggests that variability between methods is more pronounced for visceral fat measurement.

This variability in VAT may represent an inherent trade-off between precision and efficiency. While manual segmentation, guided by operator expertise, can achieve high reproducibility [14], it is impractical for large-scale applications. IACC, by providing rapid and consistent analysis, offers significant scalability advantages, even if precision for skeletal muscle is slightly compromised.

### Strengths and limitations

Despite its significant findings, this study has limitations inherent to its design. The retrospective nature of the sample while enhancing external validity by replicating the variability of routine clinical practice, restricted full control over imaging parameters, such as acquisition settings and the presence of artifacts. Although scans with obvious artifacts were excluded, subtle variations in image quality and acquisition technique may have influenced the results. Furthermore, since the sample was derived from a single center, it may not fully capture patient diversity, potentially limiting generalizability to populations with different demographic or disease profiles.

Nevertheless, the study has strong methodological points. Validation of manual segmentation by an experienced radiologist ensures that the reference method was of high quality and reliability. The excellent Dice coefficients obtained in inter-rater analysis (SAT: 0.982; MUSCLE: 0.940; VAT: 0.932) confirm that the trained evaluator accurately reproduced expert-level segmentation, solidifying the foundation for comparison with IACC.

Still, the greater bias and wider dispersion of values observed in skeletal muscle measurements highlight a notable limitation of the tool for this specific compartment.

## Conclusion

This study demonstrated that IACC is a promising and feasible tool for automated body composition (BC) analysis from CT scan. The findings confirm that for skeletal muscle and subcutaneous adipose tissue (SAT) segmentation, IACC provides high agreement and negligible bias compared with 3D Slicer, whose manual segmentation method was validated by an experienced radiologist. This methodological validation strengthens the reliability of our reference standard and, consequently, reinforces the robustness of the observed agreement.

In conclusion, IACC demonstrated high accuracy and efficiency for automated segmentation of SAT and VAT, with negligible bias. While high agreement was observed across all tissues, skeletal muscle exhibited the greatest bias and widest dispersion, identifying it as the primary source of disagreement between automated and manual methods. Despite this, the operational efficiency of IACC—reducing analysis time from approximately 30 min to just 5 min—enables a substantial gain in scalability. Consequently, IACC emerges as a strategic tool for population-based research and clinical data reuse, where speed and standardization are paramount, though caution is advised when interpreting skeletal muscle quantification until further refinements mitigate current biases. As next steps, external validation of IACC in a larger and more diverse patient cohort is imperative to confirm its generalizability. Investigating the underlying causes of variability in VAT, possibly through in-depth comparative analyses of segmentation algorithms, may provide the path to refine the tool’s accuracy, consolidating it as a fully viable and reliable solution across all compartments.

## Methods

This retrospective cross-sectional study was conducted with CT images acquired between November 2023 and January 2025 at the Centro de Imagem Diagnóstica (CIMAD), in Campo Largo, Paraná, Brazil. In accordance with the principles of the Declaration of Helsinki, the Research Ethics Committee of UTFPR via Plataforma Brasil, reviewed and approved the study protocol, including the waiver of informed consent, given the retrospective design and exclusive use of anonymized data (Approval Number 5.036.297). All CT scans were performed on a Revolution ACT GANTRY multidetector scanner (General Electric Healthcare, serial number E6WG19000029, GE Healthcare, Chicago, Illinois, USA). Initially, all images were anonymized using the clinic’s PACS system and subsequently anonymized again with the DicomAnonym MFC Application to ensure complete removal of sensitive metadata.

A total of 129 abdominal CT scans from adult participants (≥ 18 years) were initially screened. Three examinations were excluded due to artifacts or incomplete visualization of the L3 level, resulting in 126 participants included in the final analysis. For each participant, a single axial CT slice at the L3 vertebral level was used for body composition analysis.

Throughout this manuscript, the term participants refers to individuals included in the study, CT scans refers to the clinical examinations acquired, and slice refers specifically to the single L3 axial image analyzed per participant. Detailed CT parameters such as kVp, mAs, slice thickness variability, voxel size, table feed, gantry rotation time, and field of view were not consistently available in all DICOM headers and, therefore, could not be systematically reported. This heterogeneity reflects standard clinical practice and, although a limitation, enhances the external validity of this study.

Sample size was calculated based on the intraclass correlation coefficient (ICC), considering an expected ICC of 0.85, confidence interval width of 0.076, and power of 80%. This resulted in a required minimum sample of 116 participants. To account for potential exclusions, a target of 129 participants was established. After exclusions due to artifacts or inadequate visualization of L3, 126 participants remained for statistical analysis. [17].

**Fig. 4:**
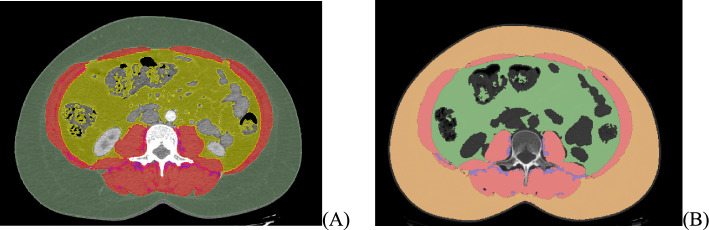
Example of body composition segmentation on the same axial L3 CT slice of a single participant. **A** Manual segmentation performed in 3D Slicer. **B** Automated segmentation performed using the IACC software

Figure 4 provides an illustrative example of the output images after segmentation of body tissues in an axial CT slice at the L3 level, comparing the manual reference method with the fully automated segmentation generated by the IACC model. This comparison highlights differences in boundary delineation between expert manual segmentation performed in 3D Slicer and the automated algorithm, underscoring the correspondence between the two approaches.

Automated segmentation of the 126 CT images was performed using the IACC software, which identified the L3 region and segmented tissue compartments based on predefined Hounsfield unit (HU) ranges. IACC is an academic research software developed in 2023 in Curitiba, Paraná, Brazil, by Computer Engineering students. It is not a commercial product and does not hold regulatory approval from agencies, such as the U.S. Food and Drug Administration (FDA) or other international authorities. Therefore, its use in this study is restricted to methodological and research validation.

Manual segmentation was performed in 3D Slicer using the Segment Editor module. For each participant, only one axial CT slice at the L3 level was segmented, and the same slice was used for automated analysis in IACC to ensure direct comparability between methods, applying thresholds of – 190 to – 30 HU for subcutaneous, visceral, and intramuscular adipose tissues (SAT, VAT, and IMAT) and – 29 to + 150 HU for skeletal muscle (MUSCLE), as previously established in the literature by Heymsfield et al. [12].

To assess intra-rater reproducibility, Evaluator 1 repeated the manual segmentation of 14 randomly selected participants (approximately 10% of the sample), with repeat measurements performed after an interval of approximately 1 month. To ensure the quality of manual segmentation, these results were also compared with independent manual segmentations performed by an experienced radiologist using the 3D Slicer.

IACC directly provides tissue area values in cm^2^, in 3D Slicer, tissue area was calculated from the number of segmented pixels and the pixel spacing contained in the DICOM metadata, using the following expression:

$$Area\,{\mkern 1mu} \left( {cm} \right){\mkern 1mu} \, = {\mkern 1mu} \,number{\mkern 1mu} \,of\,{\mkern 1mu} pixels{\mkern 1mu} \, \times \,{\mkern 1mu} \left( {pixel{\mkern 1mu} spacing{\mkern 1mu} in{\mkern 1mu} mm} \right){\mkern 1mu} \, \div \,{\mkern 1mu} 100$$

where division by 100 converts mm^2^ to cm^2^. The pixel spacing was used only to convert pixel counts into area measurements, and not to describe voxel geometry across the CT volume.

The measurements from the 126 segmentations generated by IACC were compared with those obtained in 3D Slicer. Specifically, skeletal muscle and adipose tissue (visceral and subcutaneous) areas provided by IACC were compared with manually obtained values, agreement between automated and manual measurements was assessed using intraclass correlation coefficient (ICC), Pearson’s correlation, and Bland–Altman analysis. Accordingly, the analyses applied for method comparison included: intra-rater reproducibility assessed by ICC, agreement between automated and manual methods (validated by inter-rater comparison), and Bland–Altman analysis to estimate bias and limits of agreement between automated and manual segmentation.

## Data Availability

The data sets generated and/or analyzed during the current study are available from the corresponding author on reasonable request.
